# Clinical Status and Salivary aMMP-8 Evaluation of 0.12% Chlorhexidine Versus MicroRepair^®^ ABX Mouthwash in the Non-Surgical Management of Plaque-Induced Gingivitis: A Randomized Controlled Trial

**DOI:** 10.3390/dj14060383

**Published:** 2026-06-20

**Authors:** Andrea Scribante, Maurizio Pascadopoli, Matteo Pellegrini, Cinzia Casu, Eva Massazzi, Andrea Butera

**Affiliations:** 1 Unit of Dental Hygiene, Section of Dentistry, Department of Clinical, Surgical, Diagnostic and Pediatric Sciences, University of Pavia, 27100 Pavia, Italy; andrea.scribante@unipv.it (A.S.); eva.massazzi01@universitadipavia.it (E.M.); andrea.butera@unipv.it (A.B.); 2 Unit of Orthodontics and Pediatric Dentistry, Section of Dentistry, Department of Clinical, Surgical, Diagnostic and Pediatric Sciences, University of Pavia, 27100 Pavia, Italy; maurizio.pascadopoli01@universitadipavia.it; 3 Section of Dentistry, Department of Clinical, Surgical, Diagnostic and Pediatric Sciences, University of Pavia, 27100 Pavia, Italy; 4 Oral Biotechnology Laboratory (OBL), Department of Molecular Biology, University of Cagliari, 09121 Cagliari, Italy; cinzia.casu2@unica.it

**Keywords:** aMMP-8, cetylpyridinium chloride, chlorhexidine, dentistry, gingivitis, honokiol, magnolol, randomized controlled trial

## Abstract

**Objectives**: To compare the adjunctive efficacy of a MicroRepair^®^ mouthwash containing an antibacterial complex (ABX), composed of cetylpyridinium chloride, magnolol, and honokiol, with 0.12% chlorhexidine (CHX) in the management of generalized plaque-induced gingivitis, assessing clinical periodontal parameters, salivary activated matrix metalloproteinase-8 (aMMP-8) levels, and patient-reported outcomes over 6 months. **Methods**: A randomized, controlled, parallel-group clinical trial included 40 systemically healthy adults with generalized gingivitis and was reported in accordance with CONSORT 2025 guidelines. Following professional oral hygiene according to the Guided Biofilm Therapy (GBT) protocol, participants were randomly allocated to ABX or 0.12% CHX, used twice daily for 14 days. Clinical parameters, including Full-Mouth Bleeding Score (FMBS, primary outcome), Full-Mouth Plaque Score (FMPS), Probing Pocket Depth (PPD), Clinical Attachment Level (CAL), Gingival Recession (REC), and Modified Lobene Stain Index (MLSI), were recorded at baseline, 2 weeks, 1, 3, and 6 months. Salivary aMMP-8 levels were assessed at baseline and 2 weeks. Heavy smokers were excluded, and smoking status was evaluated as a potential covariate. Non-parametric tests were applied (*p* < 0.05). **Results**: Both groups showed significant reductions in FMBS and FMPS over time (*p* < 0.05), with no intergroup differences for the primary outcome at any follow-up at the patient level. Patient-level analyses did not reveal consistent differences across secondary parameters. At the tooth level, lower FMPS values were observed in the trial group at 2 weeks and 1 month (*p* < 0.05), with earlier PPD reduction. CAL, and REC remained stable. Salivary aMMP-8 levels decreased significantly in both groups without intergroup differences. Patient-reported outcomes were comparable. Smoking status was balanced between groups and was not significantly associated with treatment allocation or the main clinical outcomes. **Conclusions**: No significant differences were observed between ABX and CHX for the main clinical and molecular outcomes, supporting its potential use as an adjunct in gingivitis management.

## 1. Introduction

Gingivitis is a reversible inflammatory condition affecting the marginal periodontal tissues, primarily induced by the accumulation of supragingival biofilm and the host immune inflammatory response to bacterial challenge [1,2]. It is clinically characterized by gingival erythema, edema, and bleeding on probing, without clinical attachment loss or radiographic evidence of alveolar bone resorption, distinguishing it from periodontitis [2,3]. According to the 2017 World Workshop classification, gingivitis can be defined and stratified based on the extent of bleeding on probing (BoP), with BoP ≥ 30% indicating generalized gingival inflammation [4].

The cornerstone of gingivitis management is the mechanical disruption and removal of dental biofilm through professional oral hygiene procedures combined with effective patient-performed plaque control [5,6,7,8,9,10]. Adjunctive use of chemical agents, particularly antimicrobial mouthwashes, has been widely recommended to enhance clinical outcomes, especially in patients with inadequate oral hygiene or high inflammatory burden [7,11]. Among these, chlorhexidine digluconate (CHX) is considered the gold standard due to its broad-spectrum antimicrobial activity, high substantivity, and proven efficacy in reducing plaque accumulation and gingival inflammation [12,13]. However, prolonged use of CHX is associated with well-documented adverse effects, including tooth staining, taste alteration, mucosal irritation, and increased calculus formation, which may limit patient compliance and long-term use [14,15,16]. Furthermore, CHX exerts a non-selective antimicrobial effect that may disrupt the ecological balance of the oral microbiota, potentially affecting microbial homeostasis [17,18].

In recent years, increasing attention has been directed toward the development of alternative therapeutic agents capable of providing effective antimicrobial and anti-inflammatory activity while improving tolerability and preserving microbial balance [19,20,21,22,23,24,25,26,27].

In this context, multi-component antibacterial systems combining different bioactive molecules have emerged as promising strategies to target oral biofilm through complementary mechanisms of action [28,29]. In particular, formulations incorporating cetylpyridinium chloride (CPC), a quaternary ammonium compound with well-documented antiseptic properties, together with natural bioactive compounds such as magnolol and honokiol, derived from Magnolia officinalis, have shown synergistic antibacterial and anti-inflammatory effects [30,31]. These compounds are known to interfere with bacterial membrane integrity, inhibit biofilm formation, and modulate inflammatory pathways, potentially enhancing plaque control while maintaining a more favorable safety profile compared with conventional antiseptics [32,33].

Within this framework, MicroRepair^®^ ABX-based formulations integrate a complex antibacterial system in which CPC, magnolol, and honokiol represent the core active components, while biomimetic hydroxyapatite primarily plays an adjunctive role related to surface interaction and enamel protection rather than being the main therapeutic driver [34,35,36,37,38,39]. Although hydroxyapatite-based systems have been widely investigated for their ability to reduce bacterial adhesion and promote remineralization [40,41,42], the clinical efficacy of these formulations in gingivitis management is likely influenced by the presence of additional antibacterial agents capable of directly modulating the oral microbiota [43].

Despite these promising characteristics, the clinical evidence supporting complex antibacterial mouthwashes combining CPC and plant-derived bioactive compounds in the management of gingivitis remains limited and fragmented [44,45]. Most available studies have focused on single-agent or remineralizing formulations rather than on multi-target antibacterial systems and often lack robust randomized controlled designs or standardized clinical endpoints [44,45,46]. Moreover, traditional clinical indices alone may not fully capture the biological dynamics of gingival inflammation, highlighting the need for integrating molecular biomarkers into clinical research [46,47].

In this context, activated matrix metalloproteinase-8 (aMMP-8) has been identified as a sensitive and early host-derived biomarker of periodontal inflammation and tissue breakdown, reflecting neutrophil-driven collagen degradation and inflammatory activity within periodontal tissues [47,48]. Although aMMP-8 has been most extensively investigated in periodontitis, its biological relevance is not limited to advanced periodontal destruction [49]. Elevated levels of aMMP-8 in oral fluids have been associated with active periodontal inflammation and have been shown to decrease following effective periodontal therapy, supporting its role as a valuable adjunctive diagnostic and monitoring tool [48,49,50,51]. In patients with gingivitis, aMMP-8 should therefore be interpreted as a biomarker of early inflammatory activation and gingival inflammatory burden, rather than as a marker of irreversible periodontal breakdown [52]. Its assessment may provide complementary information to conventional clinical indices, particularly bleeding on probing, by capturing the molecular response of gingival tissues to plaque-control interventions. The incorporation of aMMP-8 assessment into clinical trials may therefore provide a more comprehensive evaluation of treatment efficacy at both clinical and molecular levels [47,50].

To date, no randomized clinical trial has directly compared the efficacy of a MicroRepair^®^ ABX-based mouthwash, characterized by a multi-component antibacterial system including cetylpyridinium chloride, magnolol, and honokiol, with that of 0.12% chlorhexidine in patients with generalized plaque-induced gingivitis, particularly integrating both conventional clinical periodontal parameters and salivary aMMP-8 assessment. Given that gingivitis represents an early and reversible inflammatory condition, the inclusion of aMMP-8 in the present study was intended to explore whether this biomarker could reflect short-term modulation of gingival inflammatory activity after adjunctive antiseptic treatment, rather than to extrapolate periodontitis-related diagnostic thresholds to a gingivitis population.

The professional oral hygiene phase was standardized using the Guided Biofilm Therapy (GBT) protocol rather than conventional scaling and polishing in order to obtain a reproducible and biofilm-oriented baseline intervention [53,54]. GBT is based on biofilm disclosure, targeted removal of disclosed supragingival biofilm, air-polishing with low-abrasive powders, and selective ultrasonic instrumentation when required [53,55]. This approach was considered appropriate for patients with plaque-induced gingivitis because the primary therapeutic target is supragingival biofilm control rather than subgingival periodontal instrumentation. Moreover, by standardizing the professional debridement phase through a structured, minimally invasive, and operator-guided protocol, the potential influence of variability in mechanical treatment was reduced, allowing a clearer assessment of the adjunctive effect of the two tested mouthwashes.

Therefore, the present randomized controlled clinical trial aimed to compare the efficacy of a MicroRepair^®^ ABX-based mouthwash with a 0.12% chlorhexidine mouthwash as adjuncts to professional oral hygiene performed according to the Guided Biofilm Therapy (GBT) protocol in patients with generalized gingivitis. Clinical outcomes included Full-Mouth Bleeding Score (FMBS), Full-Mouth Plaque Score (FMPS), Probing Pocket Depth (PPD), Gingival Recession (REC), and Clinical Attachment Level (CAL), along with the evaluation of extrinsic staining using the Modified Lobene Stain Index (MLSI). Although gingivitis is not associated with clinical attachment loss by definition, CAL was recorded to confirm the absence of periodontitis at baseline, to ensure the periodontal stability of the enrolled population during follow-up, and to document that the observed changes were limited to reversible inflammatory parameters rather than to attachment-level modifications. Therefore, CAL was not considered a therapeutic target in this gingivitis population, but rather a diagnostic and safety-related periodontal parameter. In addition, salivary levels of aMMP-8 were assessed to investigate the molecular response to treatment.

The first null hypothesis is that there is no significant difference between the MicroRepair^®^ ABX mouthwash and 0.12% chlorhexidine in reducing clinical periodontal parameters over time.

The second null hypothesis is that there is no significant difference between the two treatments in reducing salivary aMMP-8 levels.

The third null hypothesis is that there is no significant difference between the two treatments in patient-reported outcomes related to tolerability, taste perception, and product acceptability.

## 2. Materials and Methods

### 2.1. Study Design

This was a monocentric, randomized, controlled, parallel-group clinical trial conducted at the Dental Clinic of the University of Pavia between May 2025 and January 2026. The study protocol was approved by the Unit Internal Review Board (approval number: 2025-0416). The trial was registered on ClinicalTrials.gov (NCT number: NCT07088653).

The study was conducted in accordance with the Declaration of Helsinki on experimentation involving human subjects, and the principles of Good Clinical Practice. The manuscript was prepared in accordance with the CONSORT 2025 checklist for RCT [56] (Table S1, Supplementary Materials). Written informed consent was obtained from all participants prior to their enrollment in the study.

### 2.2. Participants

All participant recruitment, clinical assessments, and data collection were conducted at the Dental Clinic of the Department of Clinical, Surgical, Diagnostic and Pediatric Sciences, University of Pavia, 27100 Pavia, Italy. Patients were consecutively selected among those referred for professional oral hygiene procedures and diagnosed with generalized gingivitis according to the 2017 World Workshop classification system, defined by BoP ≥ 30%, calculated as FMBS [4,57].

### 2.3. Eligibility Criteria for Participants

To ensure the homogeneity of the study population, specific inclusion and exclusion criteria were applied. Eligible participants were adults aged between 18 and 70 years, in good general health (ASA I or II), diagnosed with generalized plaque-induced gingivitis according to the 2017 World Workshop classification, defined by BoP ≥ 30%, calculated as FMBS, in the absence of clinical attachment loss, with PPD ≤ 3 mm at ≥90% of sites. Inclusion further required the presence of at least 20 natural teeth, provision of signed written informed consent, and willingness to comply with the study protocol and attend all scheduled follow-up visits.

Exclusion criteria comprised the presence of periodontitis, defined as interdental clinical attachment loss (CAL) at ≥2 non-adjacent teeth, systemic conditions known to affect periodontal status, including diabetes mellitus and immunodeficiencies, antibiotic or anti-inflammatory therapy within the previous 3 months, and professional dental prophylaxis within the previous 3 months. Additional exclusion criteria included pregnancy or breastfeeding, known allergy to CHX (Profar, FederFARMA.CO S.p.A, Carpiano, Italy) or to any component of the MicroRepair^®^ ABX formulation (Coswell S.p.A., Bologna, Italy), including cetylpyridinium chloride, magnolol, or honokiol, use of orthodontic appliances or removable prostheses, smoking more than 10 cigarettes per day, and participation in other clinical trials within the previous 6 months. The threshold of more than 10 cigarettes per day was selected to exclude heavy smokers, in whom smoking-related suppression of gingival bleeding and modification of the gingival inflammatory response may represent a major confounding factor. Conversely, light-to-moderate smokers were not excluded in order to preserve the clinical representativeness of the study population, since complete exclusion of all smokers would have reduced the external validity of the trial.

### 2.4. Clinical Procedure and Outcomes

All clinical procedures were carried out in accordance with the inclusion and exclusion criteria described above. At baseline (T0), a trained operator used a calibrated periodontal probe (UNC probe 15; Hu-Friedy, Chicago, IL, USA) to evaluate patients and to record the following clinical parameters at both the patient level and tooth level, on six sites per tooth (mesiobuccal, buccal, distobuccal, mesiolingual, lingual, distolingual): FMBS [58], FMPS [59], PPD [3], CAL [60], REC [61], and MLSI [62]. In addition, salivary levels of aMMP-8 were assessed, and patient-reported outcomes were collected through a structured questionnaire composed of 10 items evaluating taste perception and product acceptability [63] (Table S2, Supplementary Materials).

The primary outcome of the study was the change in FMBS from baseline (T0) to the follow-up time points, assessed at the patient level. Secondary outcomes included changes in FMPS, PPD, CAL, REC, MLSI, salivary aMMP-8 levels, and questionnaire responses across the same time points. At baseline, smoking status was also recorded as a dichotomous variable, distinguishing non-smokers from light-to-moderate smokers, defined as individuals smoking up to 10 cigarettes per day. This information was collected because smoking may influence gingival bleeding tendency and BoP/FMBS values and was therefore considered as a potential covariate in the additional analyses.

For the analysis at the patient level, mean values for PPD, CAL, and REC were calculated by averaging all measurements collected across all teeth. FMBS and FMPS were expressed as percentages, representing the proportion of sites with bleeding or plaque relative to the total number of examined sites per patient. MLSI was recorded as mean scores per patient.

At the tooth level, all clinical parameters were recorded at six sites per tooth (mesiobuccal, buccal, distobuccal, mesiolingual, lingual, distolingual). PPD, CAL, and REC were documented in millimeters per site, while FMBS and FMPS were recorded dichotomously at each site and subsequently expressed as percentages. MLSI was assessed per tooth.

Clinical assessments were performed at five time points: T0 (baseline), T1 (2 weeks), T2 (1 month), T3 (3 months), and T4 (6 months). Salivary samples for aMMP-8 analysis were collected at T0 and T1 using a standardized protocol and analyzed through an immunoenzymatic assay (aMMP-8 Test^®^, Biomolecular Diagnostic S.r.l., Florence, Italy) according to the manufacturer’s instructions. This timing was chosen because aMMP-8 was planned as an early secondary molecular endpoint aimed at evaluating the short-term inflammatory modulation occurring during the initial 14-day active phase of the assigned adjunctive mouthwash protocol, after a standardized professional biofilm-control intervention. Therefore, the T0–T1 comparison was intended to capture the early biological response associated with the first course of CHX or ABX use, rather than the long-term molecular trajectory over the entire 6-month clinical follow-up. After T1, clinical follow-up was primarily intended to monitor the stability of clinical periodontal outcomes over time. Moreover, later aMMP-8 measurements could have been influenced by the standardized follow-up maintenance procedures, including repeated professional supragingival debridement, reinforcement of oral hygiene instructions, and repeated use of the assigned mouthwash protocol, making them less suitable for isolating the early molecular response to the initial assigned mouthwash course alone.

After eligibility confirmation and baseline clinical assessment, participants were randomized before the initiation of any treatment procedure, including the GBT protocol, and before receiving any mouthwash. All patients subsequently underwent professional oral hygiene according to a standardized GBT protocol. The protocol included plaque disclosure to visualize supragingival biofilm, individualized oral hygiene instructions based on the disclosed plaque distribution, supragingival biofilm removal using air-polishing with erythritol powder, and selective removal of residual hard deposits using a piezoelectric ultrasonic scaler when required. At the end of the procedure, the treated surfaces were checked to confirm biofilm and calculus removal, and final rinsing/irrigation was performed. The same professional protocol was applied in both groups before the assigned domiciliary mouthwash regimen. According to the randomized allocation, the trial group received domiciliary use of a MicroRepair^®^ ABX-based mouthwash, characterized by a multi-component antibacterial system including cetylpyridinium chloride, magnolol, and honokiol, while the control group used a 0.12% chlorhexidine mouthwash. Both products were administered twice daily for 14 days, using 10 mL for 30 s without rinsing for one hour after application. The 14-day regimen was selected to reflect a short-term adjunctive antiseptic protocol following professional biofilm control, rather than continuous long-term mouthwash use. This duration is commonly adopted for chlorhexidine-based protocols in order to obtain an early antimicrobial and anti-inflammatory effect while limiting the risk of adverse effects associated with prolonged use, including staining, taste alteration, mucosal irritation, and potential disturbance of the oral microbiota. Therefore, the aim of the intervention phase was to evaluate the early adjunctive effect of the assigned mouthwash after GBT, whereas the subsequent follow-up visits were intended to monitor the persistence and stability of clinical outcomes over time.

At each follow-up visit, clinical parameters were re-evaluated in all participants. Professional supragingival debridement, reinforcement of oral hygiene instructions, and repetition of the assigned domiciliary mouthwash protocol for an additional 14 days were performed in all patients according to the same standardized follow-up schedule. Therefore, the number of professional hygiene sessions and mouthwash cycles was fixed and identical across participants and treatment groups. Patient-reported outcomes related to taste alteration were assessed at follow-up visits using a validated questionnaire composed of 10 items.

All procedures were performed by the same trained operator to ensure consistency and reproducibility of the clinical protocol.

The composition of the two tested mouthwashes is detailed in Table 1.

### 2.5. Sample Size Calculation

The sample size was calculated based on a continuous bleeding-related clinical outcome assessed in patients with gingivitis receiving adjunctive mouthwash therapy after professional mechanical plaque removal. The calculation was based on data reported by Yaneva et al. [64], who conducted a randomized controlled clinical trial comparing different mouthwash formulations in the adjunctive treatment of gingivitis. In that study, the bleeding index at the final follow-up was 17.58 ± 10.51 in the placebo mouthwash group and 11.36 ± 6.06 in the group treated with a mouthwash containing essential oils combined with 0.12% chlorhexidine. Based on these values, the expected between-group difference was 6.22, with a pooled standard deviation of 8.58, corresponding to an expected standardized effect size of 0.73. A two-sided significance level (alpha) of 0.05 and a statistical power of 90% were set.

Based on these assumptions, a total sample size of 40 participants was determined to be sufficient to detect a statistically significant difference between the two groups, corresponding to 20 participants per group.

### 2.6. Randomization

Using a block randomization procedure, the data analyst generated a randomization sequence with permuted blocks in R software (version 4.5.1; R Foundation for Statistical Computing, Vienna, Austria) using the blockrand package. A total of 40 participants were randomly assigned in a 1:1 ratio to either the trial or control group. Randomization was performed after eligibility confirmation and baseline clinical recordings, and before any professional treatment procedure, including the GBT protocol, or domiciliary mouthwash use. Therefore, the chronological sequence of the study procedures was as follows: eligibility confirmation, baseline clinical and salivary recordings, random allocation, standardized GBT professional treatment, and delivery of the assigned mouthwash. Allocation was concealed using sequentially numbered, opaque, sealed envelopes, prepared by a third clinician not involved in the study and opened only at the time of assignment.

Participants in the trial group received professional oral hygiene according to the GBT protocol combined with domiciliary use of a MicroRepair^®^ ABX-based mouthwash (Coswell S.p.A., Bologna, Italy), characterized by a multi-component antibacterial system including cetylpyridinium chloride, magnolol, and honokiol, applied twice daily for 14 days, using 10 mL for 30 s without rinsing for one hour after application.

Participants in the control group received the same professional oral hygiene protocol combined with a 0.12% chlorhexidine mouthwash (Profar, FederFARMA.CO S.p.A, Carpiano, Italy), administered with the same modality and duration. Participants were instructed to follow the assigned regimen precisely, and no additional domiciliary treatments were introduced.

### 2.7. Blinding and Calibration

For the domiciliary protocols, the two products (MicroRepair^®^ ABX-based mouthwash and 0.12% chlorhexidine mouthwash) were supplied in their original, clearly labeled packaging, allowing participants to follow the assigned regimen correctly. Due to differences in formulation and taste, blinding of participants was not feasible. Group allocation and product instructions were managed by a clinician not involved in clinical measurements, in accordance with the allocation concealment procedure.

The trained clinical examiner responsible for periodontal assessments (FMBS, FMPS, PPD, CAL, REC, and MLSI) was not involved in the randomization process and remained unaware of group allocation throughout the study. This was ensured by assigning product delivery and instructions to a separate clinician and performing all clinical measurements independently. In addition, the statistician performing data analysis was not involved in the clinical procedures and remained unaware of group allocation during the statistical analysis phase.

Prior to the start of the study, the examiner underwent a calibration process on ten patients not included in the trial, each presenting at least one site with a PPD ≥ 4 mm. Periodontal parameters were recorded on two separate occasions, 48 h apart. Calibration was considered successful when at least 90% of repeated measurements showed a discrepancy of less than 1 mm, ensuring a high level of intra-examiner reliability and methodological consistency [65].

### 2.8. Statistical Analysis

Statistical analyses were performed using R software (version 4.5.1; R Foundation for Statistical Computing, Vienna, Austria). Data were evaluated at both the tooth level and patient level. Descriptive statistics, including means, standard deviations, medians, ranges, and 95% confidence intervals, were calculated for each clinical parameter within the two study groups.

The normality of data distribution was assessed using the Kolmogorov–Smirnov test. Given the non-parametric nature of the data, within-group comparisons over time and between-group comparisons at each time point were performed using the Friedman test for repeated measures. In case of statistically significant differences, Dunn’s post hoc test was applied for pairwise multiple comparisons. A letter-based grouping method was used to facilitate the interpretation of statistically significant differences across comparisons [66].

Considering the potential influence of smoking on gingival bleeding response, smoking status was additionally evaluated as a potential covariate. Heavy smokers, defined as individuals smoking more than 10 cigarettes per day, were excluded according to the eligibility criteria, whereas light-to-moderate smokers were included to preserve the clinical representativeness of the study population. Additional regression-based analyses were performed to assess the association between smoking status, treatment allocation, time, and the main clinical periodontal outcomes, including FMBS, FMPS, PPD, CAL, REC, and MLSI. These analyses were conducted to verify whether the inclusion of light-to-moderate smokers could have influenced the interpretation of the primary and secondary clinical outcomes.

The level of statistical significance was set at *p* < 0.05 for all tests.

## 3. Results

### 3.1. Participants and Recruitment

**Figure 1:**
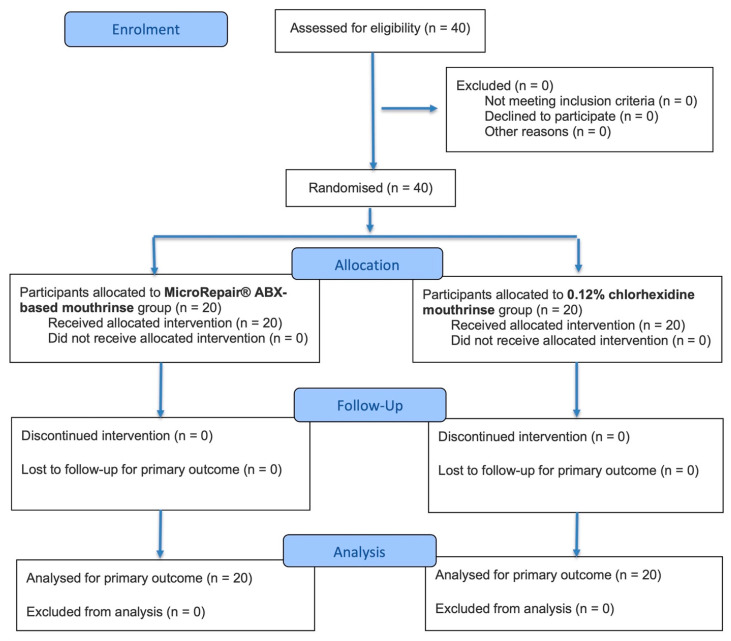
CONSORT 2025 flow diagram.

Figure 1 shows the CONSORT 2025 flow diagram of the study [56]. After screening, 40 patients fulfilling the inclusion criteria were recruited at the Unit of Dental Hygiene, Section of Dentistry, Department of Clinical, Surgical, Diagnostic and Pediatric Sciences, University of Pavia, Italy. Recruitment began in May 2025 and was completed in January 2026.

Participants were randomly allocated in a 1:1 ratio into two parallel groups: 20 patients were assigned to the trial group (MicroRepair^®^ ABX-based mouthwash), and 20 to the control group (0.12% chlorhexidine mouthwash).

All participants completed the scheduled follow-up visits at T1 (2 weeks), T2 (1 month), T3 (3 months), and T4 (6 months). No patients discontinued the intervention or were lost to follow-up. Therefore, no attrition occurred during the study period, and the final analyzed sample corresponded to the randomized sample. For this reason, no additional recruitment to compensate for dropouts was required. Participant retention was facilitated by scheduling follow-up visits in advance, providing reminders before each appointment, and maintaining direct contact with participants throughout the study period. Importantly, no adverse events or harms related to the use of either mouthwash were reported during the study period.

### 3.2. Demographic Characteristics

The demographic characteristics of the study population are summarized in Table 2. A total of 40 participants were included, comprising 23 females (57.5%) and 17 males (42.5%). The overall mean age was 35.4 ± 15.6 years, with values ranging from 18 to 65 years. In the trial group, the mean age was 33.8 ± 15.8 years (range: 19 to 64 years), while in the control group it was 37.0 ± 15.4 years (range: 18 to 65 years).

Regarding country of origin, the majority of participants were from Italy (80%), followed by Romania (10%), Morocco (5%), and Albania (5%). The distribution across groups was comparable, with participants from Italy equally represented in both groups (80%). Participants from Morocco were present only in the trial group (10%), whereas participants from Albania were observed only in the control group (10%).

Smoking status was evenly distributed between groups, with 20 participants (50%) identified as light-to-moderate smokers and 20 (50%) as non-smokers, corresponding to 10 smokers and 10 non-smokers in each group. No heavy smokers, defined as individuals smoking more than 10 cigarettes per day, were included. Because smoking may suppress gingival bleeding and influence BoP/FMBS values, this balanced distribution was considered when interpreting bleeding-related outcomes. No participants reported systemic diseases or the use of medications known to influence periodontal conditions.

### 3.3. Outcomes

Given the known effect of smoking on gingival bleeding tendency, FMBS results were interpreted in light of the balanced distribution of light-to-moderate smokers between groups and the exclusion of heavy smokers. To further assess the potential influence of smoking on the clinical outcomes, additional regression-based analyses were performed. Smoking status was not significantly associated with treatment allocation (estimate = −0.101, standard error = 0.113, *p* = 0.375), confirming that smokers and non-smokers were not unevenly distributed between the two study arms. Moreover, smoking status was not significantly associated with FMBS (estimate = −0.236, standard error = 0.251, *p* = 0.350), FMPS (estimate = −0.039, standard error = 0.122, *p* = 0.749), PPD (estimate = −0.291, standard error = 0.188, *p* = 0.126), CAL (estimate = −0.271, standard error = 0.319, *p* = 0.398), REC (estimate = −0.778, standard error = 0.425, *p* = 0.071), MLSI (estimate = 0.424, standard error = 0.433, *p* = 0.331), or time (estimate < 0.001, standard error = 0.028, *p* = 1.000). These findings suggest that the inclusion of light-to-moderate smokers did not materially affect the interpretation of the primary or secondary clinical outcomes. The complete regression-based assessment of smoking status as a potential covariate is reported in Table S3 (Supplementary Materials).

At the tooth level, the mean FMBS in the control group decreased from 0.36 ± 0.48 (95% CI: 0.14–0.58) at baseline (T0) to 0.03 ± 0.16 (95% CI: −0.04–0.10) at 6 months (T4). In the trial group, a marked reduction was observed, from 0.48 ± 0.50 (95% CI: 0.25–0.71) at T0 to 0.00 ± 0.06 (95% CI: −0.03–0.03) at T4. Intragroup analysis showed that, in the control group, T0 values were significantly higher than all subsequent time points (all *p* < 0.0001), with a progressive reduction observed at T1 and a further significant decrease at T2, after which values remained stable through T3 and T4. In the trial group, T0 values were significantly higher than T1, T2, T3, and T4 (*p* < 0.0001), and T1 was significantly higher than T2, T3, and T4 (*p* < 0.0001), while no significant differences were found among T2, T3, and T4. Intergroup comparisons revealed a statistically significant difference at baseline, with higher FMBS values in the trial group (*p* < 0.0001), whereas no statistically significant differences were detected between groups at subsequent time points. At the patient level, the mean FMBS in the control group decreased significantly from 0.36 ± 0.09 (95% CI: 0.32–0.40) at T0 to 0.03 ± 0.03 (95% CI: 0.02–0.04) at T4. In the trial group, values decreased significantly from 0.48 ± 0.15 (95% CI: 0.41–0.55) at baseline to 0.00 ± 0.00 (95% CI: 0.00–0.00) at 6 months (*p* < 0.0001). Intragroup analysis showed that, in the control group, T2, T3, and T4 were significantly lower than T0 (*p* = 0.0062 for T2; *p* < 0.0001 for T3 and T4), while T1 did not differ significantly from baseline. In the trial group, T2, T3, and T4 were significantly lower than T0 (*p* < 0.0001), whereas T1 showed no statistically significant difference. Intergroup comparisons at each time point demonstrated no statistically significant differences between the two groups. Comprehensive descriptive data and statistical comparisons are reported in Table 3.

At the tooth level, the mean FMPS in the control group decreased from 0.91 ± 0.29 (95% CI: 0.78–1.04) at baseline (T0) to 0.04 ± 0.19 (95% CI: −0.04–0.12) at 6 months (T4). In the trial group, values decreased from 0.85 ± 0.36 (95% CI: 0.69–1.01) at T0 to 0.00 ± 0.03 (95% CI: −0.01–0.01) at T4. Intragroup analysis showed that, in the control group, FMPS values decreased significantly over time, with T1 significantly lower than T0 and a further significant reduction at T2 (*p* < 0.0001), while T3 and T4 showed a further slight decrease compared with T2 and remained significantly lower than baseline (*p* < 0.0001). In the trial group, a similar trend was observed, with T0 significantly higher than T1, T2, T3, and T4 (*p* < 0.0001), and T1 significantly higher than T2, T3, and T4 (*p* < 0.0001), whereas no significant differences were found among T2, T3, and T4. Intergroup comparisons revealed no statistically significant differences at baseline, whereas lower FMPS values were observed in the trial group at T1 (*p* < 0.0001) and T2 (*p* = 0.0156); however, these differences were not maintained at later time points and were not confirmed at the patient level. No statistically significant differences between groups were detected at T3 and T4. At the patient level, the mean FMPS in the control group decreased from 0.91 ± 0.21 (95% CI: 0.81–1.01) at T0 to 0.03 ± 0.03 (95% CI: 0.02–0.04) at T4. In the trial group, values decreased from 0.85 ± 0.26 (95% CI: 0.73–0.97) at baseline to 0.00 ± 0.00 (95% CI: 0.00–0.00) at 6 months. Intragroup analysis indicated that, in the control group, T2, T3, and T4 were significantly lower than T0 (*p* = 0.0015 for T2; *p* < 0.0001 for T3 and T4), while T1 did not differ significantly from baseline. In the trial group, T2, T3, and T4 were significantly lower than T0 (*p* < 0.0001), whereas T1 showed no statistically significant difference. Intergroup comparisons at the patient level revealed no statistically significant differences at any time point. Comprehensive descriptive data and statistical comparisons are reported in Table 4.

At the tooth level, the mean PPD in the control group showed a slight reduction from 3.28 ± 0.46 mm (95% CI: 3.06–3.50) at baseline (T0) to 3.09 ± 0.28 mm (95% CI: 2.96–3.22) at 6 months (T4). In the trial group, an earlier reduction was observed, with values decreasing from 3.42 ± 0.66 mm (95% CI: 3.11–3.73) at T0 to 2.89 ± 0.35 mm (95% CI: 2.73–3.05) at T4. Intragroup analysis showed that, in the control group, T0 and T1 values were not significantly different, while a significant reduction was observed from T2 onward, with T2, T3, and T4 all significantly lower than baseline (*p* < 0.0001) and not significantly different from each other. In the trial group, a significant reduction was already evident at T1 compared with T0 (*p* < 0.0001), and all subsequent time points (T2, T3, and T4) remained significantly lower than baseline (*p* < 0.0001), without further significant changes over time. Intergroup comparisons at the tooth level revealed statistically significant differences (*p* < 0.0001), with lower PPD values in the trial group; however, these differences should be interpreted in light of the analytical level and the lack of consistent confirmation at the patient level. At the patient level, the mean PPD in the control group decreased from 3.28 ± 0.18 mm (95% CI: 3.20–3.36) at T0 to 3.09 ± 0.10 mm (95% CI: 3.05–3.13) at T4. In the trial group, values decreased from 3.50 ± 0.31 mm (95% CI: 3.36–3.64) at baseline to 2.91 ± 0.16 mm (95% CI: 2.84–2.98) at 6 months. Intragroup analysis showed that, in the control group, T2, T3, and T4 were significantly lower than T0 (*p* = 0.0451 and *p* = 0.0017), while T1 did not differ significantly from baseline. In the trial group, all follow-up time points (T1, T2, T3, and T4) were significantly lower than T0 (*p* < 0.0001), with no significant differences among them. Intergroup comparisons at the patient level indicated a statistically significant difference at T1 (*p* = 0.0281), whereas no statistically significant differences were observed at baseline or at subsequent follow-up time points. Comprehensive descriptive data and statistical comparisons are reported in Table 5.

At the tooth level, the mean CAL in the control group remained stable over time, with values of 3.10 ± 0.31 mm (95% CI: 2.95–3.25) at baseline (T0) and 3.10 ± 0.31 mm (95% CI: 2.95–3.25) at 6 months (T4). Similarly, in the trial group, CAL values showed minimal variation, ranging from 2.94 ± 0.83 mm (95% CI: 2.55–3.33) at T0 to 2.91 ± 0.39 mm (95% CI: 2.73–3.09) at T4. Intragroup analysis demonstrated no statistically significant differences across time points in either group, indicating the absence of measurable changes in CAL throughout the follow-up period. Intergroup comparisons indicated no statistically significant difference at baseline, whereas lower CAL values were observed in the trial group from T1 to T4 (*p* < 0.0001); however, these differences were not confirmed at the patient level. At the patient level, CAL values remained unchanged in both groups across all time points. In the control group, the mean CAL was 3.10 ± 0.11 mm (95% CI: 3.05–3.15) at baseline and remained identical at all subsequent follow-ups. In the trial group, the mean CAL was 2.94 ± 0.20 mm (95% CI: 2.85–3.03) at T0 and remained stable throughout the study period. Intragroup analysis confirmed the absence of statistically significant changes over time in both groups. Intergroup comparisons showed no statistically significant differences at any time point. Comprehensive descriptive data and statistical comparisons are reported in Table 6.

At the tooth level, the mean REC in the control group remained stable over time, with values of 0.10 ± 0.40 mm (95% CI: −0.08–0.28) at baseline (T0) and 0.08 ± 0.37 mm (95% CI: −0.09–0.25) at 6 months (T4). Similarly, in the trial group, REC values showed no variation, with a mean of 0.11 ± 0.44 mm (95% CI: −0.10–0.32) at T0 that remained unchanged across all follow-up time points. Intragroup analysis demonstrated no statistically significant differences over time in either group. At the patient level, REC values also remained constant throughout the study period in both groups. In the control group, the mean REC was 0.05 ± 0.09 mm (95% CI: 0.01–0.09) at baseline and remained unchanged at all subsequent follow-ups. In the trial group, the mean REC was 0.11 ± 0.16 mm (95% CI: 0.04–0.18) at T0 and remained stable over time. Intragroup analysis confirmed the absence of statistically significant changes across all time points in both groups, and intergroup comparisons showed no statistically significant differences at any time point. Comprehensive descriptive data and statistical comparisons are reported in Table 7.

At the tooth level, the mean MLSI in the control group decreased from 0.11 ± 0.31 (95% CI: −0.03–0.25) at baseline (T0) to 0.01 ± 0.10 (95% CI: −0.04–0.06) at 6 months (T4). In the trial group, values decreased from 0.22 ± 0.41 (95% CI: 0.03–0.41) at T0 to 0.04 ± 0.20 (95% CI: −0.05–0.13) at T4. Intragroup analysis showed that, in the control group, T2, T3, and T4 were significantly lower than T0 (*p* = 0.0042), while T0 and T1 were not significantly different. In the trial group, T2, T3, and T4 were significantly lower than T0 (*p* < 0.0001), whereas T0 and T1 did not differ significantly, and no further differences were observed among the later time points. Intergroup comparisons revealed statistically significant differences at T0 (*p* = 0.0017) and T1 (*p* = 0.002), with higher MLSI values in the trial group; however, these differences were not observed at later follow-up time points or at the patient level. At the patient level, the mean MLSI in the control group decreased from 0.11 ± 0.11 (95% CI: 0.06–0.16) at T0 to 0.01 ± 0.04 (95% CI: −0.01–0.03) at T4. In the trial group, values decreased from 0.21 ± 0.17 (95% CI: 0.13–0.29) at baseline to 0.04 ± 0.05 (95% CI: 0.02–0.06) at 6 months. Intragroup analysis indicated that, in the control group, T2, T3, and T4 were significantly lower than T0 (*p* = 0.0104), while T1 did not differ significantly from baseline. In the trial group, no statistically significant differences were observed across time points. Intergroup comparisons at the patient level revealed no statistically significant differences at any time point. Comprehensive descriptive data and statistical comparisons are reported in Table 8.

The salivary levels of aMMP-8 showed a marked reduction from baseline to the 2-week follow-up in both groups (Table 9). In the control group, mean aMMP-8 values decreased from 95.85 ± 48.84 (95% CI: 74.92–116.78) at baseline (T0) to 45.80 ± 41.45 (95% CI: 28.03–63.57) at T1. In the trial group, a more pronounced reduction was observed, with values decreasing from 104.50 ± 38.15 (95% CI: 88.12–120.88) at T0 to 32.30 ± 27.51 (95% CI: 20.46–44.14) at 2 weeks. Intragroup analysis demonstrated a statistically significant reduction in aMMP-8 levels from baseline to T1 in both the control group (*p* = 0.0004) and the trial group (*p* < 0.0001). Intergroup comparisons revealed no statistically significant differences between groups at baseline or at the 2-week follow-up. Comprehensive descriptive data and statistical comparisons are reported in Table 9.

Patient-reported outcomes collected through the structured questionnaire (Q1–Q10), addressing perceived gingival bleeding reduction (Q1), gingival soreness (Q2), breath improvement (Q3), dentinal hypersensitivity (Q4), gingival redness (Q5), oral freshness (Q6), tooth surface smoothness (Q7), taste acceptability (Q8), tooth staining (Q9), and adverse effects or discomfort (Q10), remained stable over time in both groups, with no statistically significant intragroup differences across follow-up visits (T1–T4) for any item (Table 10). For Q1, the trial group consistently showed higher scores, ranging from 2.55 ± 0.60 (95% CI: 2.28–2.82) at T1 to 2.65 ± 0.49 (95% CI: 2.43–2.87) at T4, compared with values between 1.65 ± 0.75 (95% CI: 1.32–1.98) and 1.90 ± 0.72 (95% CI: 1.58–2.22) in the control group, without statistically significant intergroup differences. For Q2, significantly lower scores were observed in the trial group at all time points, with values of 0.45 ± 0.69 (95% CI: 0.15–0.75), compared with 1.45 ± 0.69 (95% CI: 1.15–1.75) in the control group (all *p* = 0.003). For Q3, the trial group showed significantly higher values, ranging from 1.70 ± 0.73 (95% CI: 1.38–2.02) to 1.75 ± 0.79 (95% CI: 1.40–2.10), whereas the control group remained stable at 0.60 ± 0.75 (95% CI: 0.27–0.93), with statistically significant intergroup differences at all follow-up visits (*p* = 0.0122 at T1; *p* = 0.0074 at T2, T3, and T4). Q4 showed identical maximum scores in both groups at all time points (4.00 ± 0.00; 95% CI: 4.00–4.00), indicating no perceived changes. For Q5, the trial group consistently reported higher scores, ranging from 2.70 ± 0.57 (95% CI: 2.45–2.95) to 2.75 ± 0.55 (95% CI: 2.51–2.99), compared with 1.70 ± 0.92 (95% CI: 1.30–2.10) in the control group, without statistically significant differences. For Q6, Q7, and Q8, both groups reported stable values over time, with no statistically significant differences between groups. Specifically, Q6 values were 0.45 ± 0.60 (95% CI: 0.19–0.71) in the control group and 1.10 ± 0.45 (95% CI: 0.90–1.30) in the trial group; Q7 values ranged from 2.25 ± 0.91 (95% CI: 1.85–2.65) to 2.30 ± 0.86 (95% CI: 1.92–2.68) in the control group and remained constant at 2.80 ± 0.41 (95% CI: 2.62–2.98) in the trial group; Q8 values were 1.70 ± 0.66 (95% CI: 1.41–1.99) in the control group and 1.85 ± 0.37 (95% CI: 1.69–2.01) in the trial group. For Q9, no changes were reported in either group, with values consistently equal to 0.00 ± 0.00 (95% CI: 0.00–0.00). Similarly, for Q10, negligible values were observed, with 0.10 ± 0.31 (95% CI: 0.00–0.24) in the control group and 0.00 ± 0.00 (95% CI: 0.00–0.00) in the trial group, without statistically significant differences. Comprehensive descriptive data and statistical comparisons are reported in Table 10.

## 4. Discussion

The present randomized controlled trial was designed to evaluate the clinical, early molecular, and patient-reported outcomes of a multi-component antibacterial mouthwash based on the MicroRepair^®^ ABX system compared with a 0.12% chlorhexidine mouthwash, when used as an adjunct to professional biofilm removal in patients with generalized plaque-induced gingivitis. The results should be interpreted within the statistical framework of the analyses performed. Therefore, the absence of statistically significant between-group differences should not be interpreted as evidence that the two mouthwashes are equivalent, non-inferior, or clinically interchangeable.

Regarding the first null hypothesis, no statistically significant between-group difference was detected for the primary patient-level outcome, FMBS, during follow-up. However, selected differences emerged in secondary analyses, particularly for early tooth-level plaque control and probing depth reduction. Therefore, the first null hypothesis was not rejected for the primary patient-level bleeding outcome, but the secondary findings should be interpreted cautiously and should not be used to infer definitive between-group differences or clinical equivalence. The second null hypothesis was not rejected, as both groups showed a significant early reduction in salivary aMMP-8 levels from T0 to T1, with no statistically significant between-group difference detected at either baseline or the 2-week follow-up. However, this finding should be interpreted only within the early molecular assessment window, because aMMP-8 was not measured beyond T1 and the study was not designed to assess long-term molecular trajectories. Regarding the third null hypothesis, patient-reported outcomes related to tolerability, taste perception, and product acceptability were generally stable over time in both groups, and most questionnaire items did not show statistically significant between-group differences. Nevertheless, significant intergroup differences were observed for selected items, including lower reported gingival soreness and greater breath improvement in the ABX group. Therefore, although the overall patient-reported outcome profile did not indicate major tolerability concerns in either group, the third null hypothesis should be interpreted cautiously and cannot be considered uniformly supported across all questionnaire domains. This interpretation is consistent with the current evidence base, in which chlorhexidine remains the conventional reference adjunct for plaque and gingivitis control, while multi-component antibacterial formulations combining synthetic antiseptics and bioactive compounds are increasingly being investigated as biologically compatible adjunctive options with promising clinical performance [12,13,16,67].

The most relevant clinical finding is the marked reduction in gingival inflammation observed in both groups, as reflected by FMBS. This result reinforces the central role of mechanical biofilm disruption as the main determinant of clinical improvement in plaque-induced gingivitis, with mouthwashes acting as adjunctive rather than stand-alone therapies. The substantial decrease in bleeding in both study arms is in line with systematic evidence showing that chlorhexidine can reduce plaque accumulation and support gingival improvement when added to mechanical oral hygiene. At the same time, no statistically significant between-group difference was detected for the primary patient-level bleeding outcome during follow-up. This finding indicates that the anti-inflammatory outcomes were not significantly different between groups under the present study conditions, but it should not be interpreted as evidence of clinical equivalence or non-inferiority. The observed clinical improvement in the ABX group remains biologically plausible, considering the potential of multi-component antibacterial systems to modulate gingival inflammation through combined antimicrobial and anti-inflammatory mechanisms, as suggested by studies investigating alternative non-chlorhexidine mouthwashes for plaque-induced gingivitis control [68,69,70].

Plaque-related outcomes showed reductions over time in both groups. At the tooth level, significantly lower FMPS values were observed in the ABX group at T1 and T2, suggesting a possible early reduction in plaque accumulation during the initial phases of follow-up. However, this finding was limited to tooth-level analyses and was not consistently confirmed at the patient level. Therefore, it should be interpreted as a secondary observation rather than as evidence of superior overall plaque control. Similarly, the absence of statistically significant patient-level differences should not be interpreted as proof that the two mouthwashes provide the same plaque-control effect. Overall, both interventions were associated with improved plaque-related parameters over time, but larger studies are needed to clarify whether the ABX formulation provides any additional clinically relevant effect on plaque control.

The probing depth findings require careful interpretation considering the characteristics of the study population. Because the trial included patients with generalized gingivitis and not periodontitis, baseline probing depths were low and major structural periodontal changes were not expected. The modest reduction in PPD observed in both groups, and the earlier decline in the trial group, are therefore more plausibly attributable to the resolution of inflammatory edema than to true periodontal attachment gain. This reading is supported by the stability of CAL and REC throughout follow-up. In other words, the clinical improvement documented here appears to reflect the reversal of inflammation rather than a modification of attachment status. This is in keeping with the current understanding of plaque-induced gingivitis as a reversible inflammatory condition and with the broader literature showing that anti-gingivitis interventions primarily affect inflammatory indices and soft tissue swelling rather than structural periodontal parameters in non-periodontitis populations [71,72,73].

The absence of meaningful changes in CAL and REC deserves explicit consideration. Stability of these variables across follow-up in both groups indicates that neither intervention altered the attachment profile of the included subjects, which is consistent with the enrollment of patients without periodontitis. From a methodological perspective, this strengthens the internal coherence of the findings, because reductions in bleeding and probing depth occurred in parallel with stable attachment-related measures.

The staining-related results are clinically relevant because one of the best-known limitations of chlorhexidine is its association with extrinsic pigmentation, especially with longer use. In the present study, MLSI values decreased over time in both groups, and questionnaire responses did not reveal any meaningful perception of increased staining. This finding is compatible with the fact that chlorhexidine-related discoloration is most clearly documented when rinses are used for four weeks or longer [14,15,74], whereas the protocol adopted here involved a short 14-day course. The lack of a clear staining signal in the chlorhexidine arm therefore appears plausible and should not be interpreted as contradictory to the established literature. At the same time, the ABX-based mouthwash did not show evident esthetic drawbacks under the present conditions, although this finding should be interpreted within the short duration of the mouthwash protocol.

A further aspect that should be considered is the presence of statistically significant intergroup differences at baseline in selected tooth-level outcomes, specifically FMBS, PPD, and MLSI. These findings do not necessarily indicate a true lack of baseline comparability between the randomized groups. Randomization was performed at the patient level, whereas these analyses were conducted at the tooth level; accordingly, variability in the distribution of site-specific measurements may generate occasional baseline differences, particularly in relatively small samples. This interpretation is supported by the fact that those differences were not consistently reproduced in the corresponding patient-level analyses, where baseline values were generally balanced, and by the balanced demographic and clinical profile of the two study groups at enrollment. Therefore, the most plausible explanation is random variation related to the analytical level rather than systematic allocation bias. The coherent intragroup improvement observed over time in both arms further supports this reading and suggests that the comparative interpretation of treatment effects remains valid [75,76].

The salivary aMMP-8 results add a valuable biological dimension to the clinical findings. aMMP-8 is increasingly regarded as a marker of active periodontal collagen breakdown and inflammatory burden, and several recent reviews and clinical studies have shown that its levels are elevated in inflammatory periodontal conditions and tend to decrease after effective therapy [77,78,79]. In the present study, both groups showed a significant reduction from baseline to 2 weeks, with no intergroup difference. This suggests that the clinical reduction in gingival inflammation was accompanied by an early decrease in molecular inflammatory activity in both groups. However, because aMMP-8 was assessed only from T0 to T1, the absence of a statistically significant between-group difference should not be interpreted as evidence of comparable long-term biological efficacy between the ABX-based formulation and chlorhexidine. Given the growing interest in integrating biomarkers into periodontal monitoring, this early molecular assessment provides complementary information to conventional clinical indices, while remaining limited to the short-term treatment phase.

The patient-reported outcomes complement the clinical and biomarker data and provide a more pragmatic view of treatment performance. Overall, both mouthwashes were well tolerated, no relevant adverse events emerged, and questionnaire scores remained stable over time. Some subjective domains appeared to favor the test formulation, particularly perceived breath improvement and reduced gingival soreness, while most other domains, including taste acceptability, oral freshness, tooth smoothness, staining, and discomfort, did not show statistically significant between-group differences. These observations should be interpreted cautiously because participant blinding was not feasible, and subjective outcomes are intrinsically more vulnerable to expectation effects. Moreover, the absence of statistically significant differences in several questionnaire domains should not be interpreted as proof of equivalent acceptability between the two products. Nevertheless, patient-reported outcomes remain clinically relevant, as chlorhexidine is known to be associated with taste disturbance and other sensory drawbacks in some users, and real-world adherence to home-care regimens depends heavily on product acceptability [13,15,80].

The present study has some strengths. It adopted a randomized parallel-group design, achieved complete follow-up, used a standardized professional protocol before allocation, and combined clinical, biological, and patient-reported outcomes.

Some limitations should also be acknowledged. First, the study was monocentric and included a relatively small sample. This may have reduced the statistical power to detect small between-group differences, particularly for selected secondary and subjective outcomes. Therefore, the absence of statistically significant between-group differences should not be interpreted as evidence of equivalence, non-inferiority, or clinical interchangeability between the two mouthwashes. Future studies with larger samples and predefined equivalence or non-inferiority margins are needed if the objective is to determine whether MicroRepair^®^ ABX can be considered clinically interchangeable with chlorhexidine.

Second, the absence of formal adjustment for multiple comparisons should be considered. Several secondary clinical outcomes, tooth-level and patient-level analyses, and repeated time-point comparisons were performed. Accordingly, statistically significant findings from secondary analyses should be interpreted cautiously, because the risk of type I error inflation cannot be excluded. These results should therefore be considered exploratory and hypothesis-generating rather than definitive evidence of treatment-specific effects.

Participant blinding was not feasible because of the different characteristics, packaging, and taste of the two mouthwashes. In addition, the limited availability of previous clinical trials on similar multi-component antibacterial mouthwashes restricts direct comparison with the existing literature and partly limits the contextual interpretation of the findings.

Another limitation is that salivary aMMP-8 was assessed only at baseline and at 2 weeks, whereas clinical periodontal parameters were followed for 6 months. This design was chosen because aMMP-8 was considered an early secondary molecular endpoint reflecting the short-term host-inflammatory response during the initial 14-day active phase of the assigned mouthwash protocol after standardized professional biofilm control. Therefore, the present findings on aMMP-8 should be interpreted only as evidence of early molecular modulation associated with the first course of adjunctive mouthwash use. They cannot be extrapolated to the long-term biological behavior of this biomarker during the entire follow-up period.

The standardized follow-up maintenance procedures should also be considered when interpreting the findings. For ethical and clinical reasons, professional supragingival debridement, reinforcement of oral hygiene instructions, and repetition of the assigned mouthwash protocol were performed during follow-up to ensure appropriate patient care and periodontal health. This approach was clinically and deontologically appropriate, but it represents a methodological limitation. The observed 6-month outcomes should therefore be interpreted as the effect of the assigned mouthwash within a standardized professional maintenance program, rather than as the isolated effect of a single initial 14-day mouthwash course. Future studies may compare single-course and repeated-course protocols to better distinguish the specific contribution of the mouthwash from that of professional maintenance procedures.

The inclusion of light-to-moderate smokers also warrants consideration as a potential confounding factor for bleeding-related outcomes. These participants represented 50% of the study population. However, heavy smokers were excluded, and light-to-moderate smokers were evenly distributed between groups, with 10 smokers in each arm. In addition, regression-based analyses showed that smoking status was not significantly associated with treatment allocation, FMBS, FMPS, PPD, CAL, REC, MLSI, or time. Therefore, although smoking remains biologically relevant because it may suppress gingival bleeding and influence BoP/FMBS values, the present analyses suggest that the inclusion of light-to-moderate smokers did not materially affect the interpretation of the primary or secondary clinical outcomes. Nevertheless, bleeding-related outcomes should still be interpreted with appropriate caution, particularly because FMBS represented the primary clinical outcome of the study.

Finally, the 14-day use protocol was selected to evaluate the short-term adjunctive effect of the assigned mouthwash after standardized professional biofilm control, rather than the effect of continuous long-term use. This choice was consistent with the common short-term use of chlorhexidine-based antiseptic protocols and was intended to limit the risk of adverse effects associated with prolonged mouthwash exposure, including staining, taste alteration, mucosal irritation, and potential disturbance of the oral microbiota. Therefore, the 6-month follow-up should be interpreted as a period for monitoring the persistence and stability of clinical outcomes after the initial active treatment phase, not as evidence regarding continuous mouthwash administration for 6 months. Future studies may specifically compare short-term, repeated, and prolonged mouthwash regimens to determine whether longer administration provides additional benefit.

## 5. Conclusions

Within the limitations of the present randomized clinical trial, both mouthwashes were associated with improvements in clinical periodontal parameters over the 6-month follow-up. However, the absence of statistically significant between-group differences should not be interpreted as evidence of equivalence, non-inferiority, or clinical interchangeability between MicroRepair^®^ ABX and 0.12% chlorhexidine.

Salivary aMMP-8 levels showed an early reduction after the initial 14-day active treatment phase, but no conclusions can be drawn regarding the long-term molecular behavior of this biomarker, as it was assessed only at baseline and at T1. Moreover, the interpretation of the findings should consider the limited sample size, the inclusion of light-to-moderate smokers, the absence of formal adjustment for multiple comparisons, and the conditional re-treatment design, which may have introduced unequal intervention exposure across participants. Although smoking status was balanced between groups and was not significantly associated with the main clinical outcomes in the additional regression-based analyses, its potential biological influence on gingival bleeding response should still be acknowledged. The 6-month follow-up reflects the monitoring of clinical stability after an initial 14-day active mouthwash phase and should not be interpreted as evidence of the effects of continuous long-term mouthwash use.

Overall, the results suggest that MicroRepair^®^ ABX may be a potential adjunctive option in the management of plaque-induced gingivitis, but these findings should be considered hypothesis-generating. Larger, adequately powered trials with predefined analytical strategies are needed to confirm the clinical relevance of any residual differences between MicroRepair^®^ ABX and chlorhexidine after standardized professional biofilm control.

## Figures and Tables

**Table 1:** Composition of the tested products.

Product	Manufacturer	Composition
MicroRepair^®^ ABX mouthwash	Coswell S.p.A. (Bologna, Italy)	Aqua, Sorbitol, Xylitol, Zinc Hydroxyapatite, Cetylpyridinium Chloride, Magnolol, Honokiol, Aroma, PEG-40 Hydrogenated Castor Oil, PVM/MA Copolymer, Cocamidopropyl Betaine, Sodium Myristoyl Sarcosinate, Sodium Methyl Cocoyl Taurate, Sodium Saccharin, Disodium Phosphate, Sodium Levulinate, Phenoxyethanol, Sodium Benzoate, Farnesol, Limonene
Chlorhexidine 0.12% mouthwash	Profar, FederFARMA.CO S.p.A, Carpiano, Italy	Aqua, Chlorhexidine Digluconate (0.12%), Poloxamer 407, Aroma, Sucralose, Phenoxyethanol, Eugenol, CI 19,140 (colorant), CI 42,090 (colorant)

**Table 2:** Baseline demographic and dental characteristics of the study population.

Variable	Total (*n* = 40)	ABX (*n* = 20)	CHX 0.12% (*n* = 20)
Age (years)	35.4 ± 15.6 (18–65)	33.8 ± 15.8 (19–64)	37.0 ± 15.4 (18–65)
Sex (F/M)	23 (57.5%)/17 (42.5%)	11 (55%)/9 (45%)	12 (60%)/8 (40%)
Nationality			
Italy	32 (80%)	16 (80%)	16 (80%)
Romania	4 (10%)	2 (10%)	2 (10%)
Morocco	2 (5%)	2 (10%)	0 (0%)
Albania	2 (5%)	0 (0%)	2 (10%)
Smoking status			
Yes	20 (50%)	10 (50%)	10 (50%)
No	20 (50%)	10 (50%)	10 (50%)
Systemic diseases	0 (0%)	0 (0%)	0 (0%)
Medication use	0 (0%)	0 (0%)	0 (0%)

**Table 3:** Descriptive statistics and intragroup/intergroup comparisons of FMBS tooth-level and patient-level values across all time points (T0–T4) in the Trial and Control groups. * Means presenting at least one identical letter are not significantly different (*p* > 0.05). Intragroup analysis considered all the time frames of the study for the comparisons (lowercase letters), while intergroup analysis considered only Control vs. Trial comparison at each time frame (uppercase letters).

Tooth Level							
Group	Time	Mean ± SD	Range	Median	C.I. (95%)	Intragroup (T0–T4) *	Intergroup (Control-Trial) *
Control (*n* = 20)	T0	0.36 ± 0.48	0.00–1.00	0.00	[0.14–0.58]	a	A
	T1	0.21 ± 0.41	0.00–1.00	0.00	[0.02–0.40]	b	A
	T2	0.08 ± 0.27	0.00–1.00	0.00	[−0.05–0.21]	c	A
	T3	0.04 ± 0.19	0.00–1.00	0.00	[−0.05–0.13]	c	A
	T4	0.03 ± 0.16	0.00–1.00	0.00	[−0.04–0.10]	c	A
Trial (*n* = 20)	T0	0.48 ± 0.50	0.00–1.00	0.00	[0.25–0.71]	a	B
	T1	0.16 ± 0.37	0.00–1.00	0.00	[−0.01–0.33]	b	A
	T2	0.04 ± 0.19	0.00–1.00	0.00	[−0.05–0.13]	c	A
	T3	0.01 ± 0.12	0.00–1.00	0.00	[−0.05–0.07]	c	A
	T4	0.00 ± 0.06	0.00–1.00	0.00	[−0.03–0.03]	c	A
**Patient Level**							
**Group**	**Time**	**Mean ± SD**	**Range**	**Median**	**C.I. (95%)**	Intragroup (T0–T4) *	Intergroup (Control-Trial) *
Control (*n* = 20)	T0	0.36 ± 0.09	0.22–0.51	0.34	[0.32–0.40]	a	A
	T1	0.20 ± 0.10	0.07–0.39	0.21	[0.15–0.25]	a, b	A
	T2	0.11 ± 0.18	0.00–0.80	0.07	[0.03–0.19]	b, c	A
	T3	0.04 ± 0.05	0.00–0.20	0.04	[0.02–0.06]	c	A
	T4	0.03 ± 0.03	0.00–0.11	0.02	[0.02–0.04]	c	A
Trial (*n* = 20)	T0	0.48 ± 0.15	0.30–0.84	0.44	[0.41–0.55]	a	A
	T1	0.15 ± 0.08	0.00–0.28	0.15	[0.11–0.19]	a, b	A
	T2	0.04 ± 0.04	0.00–0.11	0.03	[0.02–0.06]	c	A
	T3	0.01 ± 0.02	0.00–0.08	0.00	[0.00–0.02]	c	A
	T4	0.00 ± 0.00	0.00–0.00	0.00	[0.00–0.00]	c	A

Abbreviations: C.I., Confidence Interval; SD, standard deviation; T0, baseline; T1, 2 weeks; T2, 1 month; T3, 3 months; T4, 6 months.

**Table 4:** Descriptive statistics and intragroup/intergroup comparisons of FMPS tooth-level and patient-level values across all time points (T0–T4) in the Trial and Control groups. * Means presenting at least one identical letter are not significantly different (*p* > 0.05). Intragroup analysis considered all the time frames of the study for the comparisons (lowercase letters), while intergroup analysis considered only Control vs. Trial comparison at each time frame (uppercase letters).

Tooth Level							
Group	Time	Mean ± SD	Range	Median	C.I. (95%)	Intragroup (T0–T4) *	Intergroup (Control-Trial) *
Control (*n* = 20)	T0	0.91 ± 0.29	0.00–1.00	1.00	[0.78–1.04]	a	A
	T1	0.47 ± 0.50	0.00–1.00	0.00	[0.24–0.70]	b	A
	T2	0.10 ± 0.30	0.00–1.00	0.00	[−0.04–0.24]	c	A
	T3	0.05 ± 0.22	0.00–1.00	0.00	[−0.05–0.15]	c, d	A
	T4	0.04 ± 0.19	0.00–1.00	0.00	[−0.04–0.12]	d	A
Trial (*n* = 20)	T0	0.85 ± 0.36	0.00–1.00	1.00	[0.69–1.01]	a	A
	T1	0.23 ± 0.42	0.00–1.00	0.00	[0.04–0.42]	b	B
	T2	0.04 ± 0.19	0.00–1.00	0.00	[−0.05–0.13]	c	B
	T3	0.00 ± 0.05	0.00–1.00	0.00	[−0.02–0.02]	c	A
	T4	0.00 ± 0.03	0.00–1.00	0.00	[−0.01–0.01]	c	A
**Patient Level**							
**Group**	**Time**	**Mean ± SD**	**Range**	**Median**	**C.I. (95%)**	Intragroup (T0–T4) *	Intergroup (Control-Trial) *
Control (*n* = 20)	T0	0.91 ± 0.21	0.14–1.00	1.00	[0.81–1.01]	a	A
	T1	0.47 ± 0.16	0.00–0.66	0.49	[0.40–0.54]	a, b	A
	T2	0.10 ± 0.08	0.00–0.35	0.09	[0.06–0.14]	c	A
	T3	0.05 ± 0.04	0.00–0.14	0.05	[0.03–0.07]	c	A
	T4	0.03 ± 0.03	0.00–0.08	0.04	[0.02–0.04]	c	A
Trial (*n* = 20)	T0	0.85 ± 0.26	0.25–1.00	1.00	[0.73–0.97]	a	A
	T1	0.22 ± 0.18	0.00–0.58	0.14	[0.14–0.30]	a, b	A
	T2	0.04 ± 0.05	0.00–0.16	0.03	[0.02–0.06]	b, c	A
	T3	0.01 ± 0.02	0.00–0.06	0.00	[0.00–0.02]	c	A
	T4	0.00 ± 0.00	0.00–0.01	0.00	[0.00–0.00]	c	A

Abbreviations: C.I., Confidence Interval; SD, standard deviation; T0, baseline; T1, 2 weeks; T2, 1 month; T3, 3 months; T4, 6 months.

**Table 5:** Descriptive statistics and intragroup/intergroup comparisons of PPD tooth-level and patient-level values across all time points (T0–T4) in the Trial and Control groups. * Means presenting at least one identical letter are not significantly different (*p* > 0.05). Intragroup analysis considered all the time frames of the study for the comparisons (lowercase letters), while intergroup analysis considered only Control vs. Trial comparison at each time frame (uppercase letters).

Tooth Level							
Group	Time	Mean ± SD	Range	Median	C.I. (95%)	Intragroup (T0–T4) *	Intergroup (Control-Trial) *
Control (*n* = 20)	T0	3.28 ± 0.46	3.00–5.00	3.00	[3.06–3.50]	a	A
	T1	3.21 ± 0.41	3.00–5.00	3.00	[3.02–3.40]	a	A
	T2	3.12 ± 0.32	3.00–4.00	3.00	[2.97–3.27]	b	A
	T3	3.09 ± 0.28	3.00–4.00	3.00	[2.96–3.22]	b	A
	T4	3.09 ± 0.28	3.00–4.00	3.00	[2.96–3.22]	b	A
Trial (*n* = 20)	T0	3.42 ± 0.66	2.00–6.00	3.00	[3.11–3.73]	a	B
	T1	2.93 ± 0.38	2.00–5.00	3.00	[2.75–3.11]	b	B
	T2	2.91 ± 0.33	2.00–4.00	3.00	[2.76–3.06]	b	B
	T3	2.89 ± 0.35	2.00–4.00	3.00	[2.73–3.05]	b	B
	T4	2.89 ± 0.35	2.00–4.00	3.00	[2.73–3.05]	b	B
**Patient Level**							
**Group**	**Time**	**Mean ± SD**	**Range**	**Median**	**C.I. (95%)**	Intragroup (T0–T4) *	Intergroup (Control-Trial) *
Control (*n* = 20)	T0	3.28 ± 0.18	3.04–3.67	3.22	[3.20–3.36]	a	A
	T1	3.21 ± 0.15	3.03–3.48	3.19	[3.14–3.28]	a, b	A
	T2	3.11 ± 0.12	3.00–3.39	3.07	[3.06–3.16]	b	A
	T3	3.09 ± 0.10	3.00–3.33	3.05	[3.05–3.13]	b	A
	T4	3.09 ± 0.10	3.00–3.33	3.05	[3.05–3.13]	b	A
Trial (*n* = 20)	T0	3.50 ± 0.31	2.95–4.05	3.45	[3.36–3.64]	a	A
	T1	2.97 ± 0.18	2.62–3.17	3.02	[2.89–3.05]	b	B
	T2	2.93 ± 0.16	2.57–3.07	3.01	[2.86–3.00]	b	A
	T3	2.91 ± 0.16	2.57–3.07	3.01	[2.84–2.98]	b	A
	T4	2.91 ± 0.16	2.57–3.07	3.01	[2.84–2.98]	b	A

Abbreviations: C.I., Confidence Interval; SD, standard deviation; T0, baseline; T1, 2 weeks; T2, 1 month; T3, 3 months; T4, 6 months.

**Table 6:** Descriptive statistics and intragroup/intergroup comparisons of CAL tooth-level and patient-level values across all time points (T0–T4) in the Trial and Control groups. * Means presenting at least one identical letter are not significantly different (*p* > 0.05). Intragroup analysis considered all the time frames of the study for the comparisons (lowercase letters), while intergroup analysis considered only Control vs. Trial comparison at each time frame (uppercase letters).

Tooth Level							
Group	Time	Mean ± SD	Range	Median	C.I. (95%)	Intragroup (T0–T4) *	Intergroup (Control-Trial) *
Control (*n* = 20)	T0	3.10 ± 0.31	3.00–6.00	3.00	[2.95–3.25]	a	A
	T1	3.10 ± 0.31	3.00–6.00	3.00	[2.95–3.25]	a	A
	T2	3.10 ± 0.31	3.00–6.00	3.00	[2.95–3.25]	a	A
	T3	3.10 ± 0.31	3.00–6.00	3.00	[2.95–3.25]	a	A
	T4	3.10 ± 0.31	3.00–6.00	3.00	[2.95–3.25]	a	A
Trial (*n* = 20)	T0	2.94 ± 0.83	2.00–6.00	3.00	[2.55–3.33]	a	A
	T1	2.92 ± 0.38	2.00–6.00	3.00	[2.74–3.10]	a	B
	T2	2.92 ± 0.38	2.00–6.00	3.00	[2.74–3.10]	a	B
	T3	2.91 ± 0.39	2.00–6.00	3.00	[2.73–3.09]	a	B
	T4	2.91 ± 0.39	2.00–6.00	3.00	[2.73–3.09]	a	B
**Patient Level**							
**Group**	**Time**	**Mean ± SD**	**Range**	**Median**	**C.I. (95%)**	Intragroup (T0–T4) *	Intergroup (Control-Trial) *
Control (*n* = 20)	T0	3.10 ± 0.11	3.00–3.36	3.05	[3.05–3.15]	a	A
	T1	3.10 ± 0.11	3.00–3.36	3.05	[3.05–3.15]	a	A
	T2	3.10 ± 0.11	3.00–3.36	3.05	[3.05–3.15]	a	A
	T3	3.10 ± 0.11	3.00–3.36	3.05	[3.05–3.15]	a	A
	T4	3.10 ± 0.11	3.00–3.36	3.05	[3.05–3.15]	a	A
Trial (*n* = 20)	T0	2.94 ± 0.20	2.50–3.18	3.01	[2.85–3.03]	a	A
	T1	2.94 ± 0.20	2.50–3.18	3.01	[2.85–3.03]	a	A
	T2	2.94 ± 0.20	2.50–3.18	3.01	[2.85–3.03]	a	A
	T3	2.94 ± 0.20	2.50–3.18	3.01	[2.85–3.03]	a	A
	T4	2.94 ± 0.20	2.50–3.18	3.01	[2.85–3.03]	a	A

Abbreviations: C.I., Confidence Interval; SD, standard deviation; T0, baseline; T1, 2 weeks; T2, 1 month; T3, 3 months; T4, 6 months.

**Table 7:** Descriptive statistics and intragroup/intergroup comparisons of REC tooth-level and patient-level values across all time points (T0–T4) in the Trial and Control groups. * Means presenting at least one identical letter are not significantly different (*p* > 0.05). Intragroup analysis considered all the time frames of the study for the comparisons (lowercase letters), while intergroup analysis considered only Control vs. Trial comparison at each time frame (uppercase letters).

Tooth Level							
Group	Time	Mean ± SD	Range	Median	C.I. (95%)	Intragroup (T0–T4) *	Intergroup (Control-Trial) *
Control (*n* = 20)	T0	0.10 ± 0.40	0.00–3.00	0.00	[−0.08–0.28]	a	A
	T1	0.08 ± 0.37	0.00–3.00	0.00	[−0.09–0.25]	a	A
	T2	0.08 ± 0.38	0.00–3.00	0.00	[−0.09–0.25]	a	A
	T3	0.08 ± 0.37	0.00–3.00	0.00	[−0.09–0.25]	a	A
	T4	0.08 ± 0.37	0.00–3.00	0.00	[−0.09–0.25]	a	A
Trial (*n* = 20)	T0	0.11 ± 0.44	0.00–3.00	0.00	[−0.10–0.32]	a	A
	T1	0.11 ± 0.44	0.00–3.00	0.00	[−0.10–0.32]	a	A
	T2	0.11 ± 0.44	0.00–3.00	0.00	[−0.10–0.32]	a	A
	T3	0.11 ± 0.44	0.00–3.00	0.00	[−0.10–0.32]	a	A
	T4	0.11 ± 0.44	0.00–3.00	0.00	[−0.10–0.32]	a	A
**Patient Level**							
**Group**	**Time**	**Mean ± SD**	**Range**	**Median**	**C.I. (95%)**	Intragroup (T0–T4) *	Intergroup (Control-Trial) *
Control (*n* = 20)	T0	0.05 ± 0.09	0.00–0.32	0.00	[0.01–0.09]	a	A
	T1	0.05 ± 0.09	0.00–0.32	0.00	[0.01–0.09]	a	A
	T2	0.05 ± 0.09	0.00–0.32	0.00	[0.01–0.09]	a	A
	T3	0.05 ± 0.09	0.00–0.32	0.00	[0.01–0.09]	a	A
	T4	0.05 ± 0.09	0.00–0.32	0.00	[0.01–0.09]	a	A
Trial (*n* = 20)	T0	0.11 ± 0.16	0.00–0.64	0.07	[0.04–0.18]	a	A
	T1	0.11 ± 0.16	0.00–0.64	0.07	[0.04–0.18]	a	A
	T2	0.11 ± 0.16	0.00–0.64	0.07	[0.04–0.18]	a	A
	T3	0.11 ± 0.16	0.00–0.64	0.07	[0.04–0.18]	a	A
	T4	0.11 ± 0.16	0.00–0.64	0.07	[0.04–0.18]	a	A

Abbreviations: C.I., Confidence Interval; SD, standard deviation; T0, baseline; T1, 2 weeks; T2, 1 month; T3, 3 months; T4, 6 months.

**Table 8:** Descriptive statistics and intragroup/intergroup comparisons of MLSI tooth-level and patient-level values across all time points (T0–T4) in the Trial and Control groups. * Means presenting at least one identical letter are not significantly different (*p* > 0.05). Intragroup analysis considered all the time frames of the study for the comparisons (lowercase letters), while intergroup analysis considered only Control vs. Trial comparison at each time frame (uppercase letters).

Tooth Level							
Group	Time	Mean ± SD	Range	Median	C.I. (95%)	Intragroup (T0–T4) *	Intergroup (Control-Trial) *
Control (*n* = 20)	T0	0.11 ± 0.31	0.00–1.00	0.00	[−0.03–0.25]	a	A
	T1	0.11 ± 0.31	0.00–1.00	0.00	[−0.03–0.25]	a	A
	T2	0.01 ± 0.10	0.00–1.00	0.00	[−0.04–0.06]	b	A
	T3	0.01 ± 0.10	0.00–1.00	0.00	[−0.04–0.06]	b	A
	T4	0.01 ± 0.10	0.00–1.00	0.00	[−0.04–0.06]	b	A
Trial (*n* = 20)	T0	0.22 ± 0.41	0.00–1.00	0.00	[0.03–0.41]	a	B
	T1	0.22 ± 0.41	0.00–1.00	0.00	[0.03–0.41]	a	B
	T2	0.04 ± 0.20	0.00–1.00	0.00	[−0.05–0.13]	b	A
	T3	0.04 ± 0.20	0.00–1.00	0.00	[−0.05–0.13]	b	A
	T4	0.04 ± 0.20	0.00–1.00	0.00	[−0.05–0.13]	b	A
**Patient Level**							
**Group**	**Time**	**Mean ± SD**	**Range**	**Median**	**C.I. (95%)**	Intragroup (T0–T4) *	Intergroup (Control-Trial) *
Control (*n* = 20)	T0	0.11 ± 0.11	0.00–0.37	0.09	[0.06–0.16]	a	A
	T1	0.11 ± 0.11	0.00–0.37	0.09	[0.06–0.16]	a	A
	T2	0.01 ± 0.04	0.00–0.18	0.00	[−0.01–0.03]	b	A
	T3	0.01 ± 0.04	0.00–0.18	0.00	[−0.01–0.03]	b	A
	T4	0.01 ± 0.04	0.00–0.18	0.00	[−0.01–0.03]	b	A
Trial (*n* = 20)	T0	0.21 ± 0.17	0.00–0.58	0.19	[0.13–0.29]	a	A
	T1	0.21 ± 0.17	0.00–0.58	0.19	[0.13–0.29]	a	A
	T2	0.04 ± 0.05	0.00–0.16	0.02	[0.02–0.06]	a	A
	T3	0.04 ± 0.05	0.00–0.16	0.02	[0.02–0.06]	a	A
	T4	0.04 ± 0.05	0.00–0.16	0.02	[0.02–0.06]	a	A

Abbreviations: C.I., Confidence Interval; SD, standard deviation; T0, baseline; T1, 2 weeks; T2, 1 month; T3, 3 months; T4, 6 months.

**Table 9:** Descriptive statistics and intragroup/intergroup comparisons of aMMP-8 values between baseline (T0) and follow-up at 2 weeks (T1) in the Trial and Control groups. * Means presenting at least one identical letter are not significantly different (*p* > 0.05). Intragroup analysis considered all the time frames of the study for the comparisons (lowercase letters), while intergroup analysis considered only Control vs. Trial comparison at each time frame (uppercase letters).

Group	Time	Mean ± SD	Range	Median	C.I. (95%)	Intragroup (T0–T1) *	Intergroup (Control-Trial) *
Control (*n* = 20)	T0	95.85 ± 48.84	35.00–182.00	91.50	[74.92–116.78]	a	A
	T1	45.80 ± 41.45	0.00–130.00	30.50	[28.03–63.57]	b	A
Trial (*n* = 20)	T0	104.50 ± 38.15	25.00–156.00	115.00	[88.12–120.88]	a	A
	T1	32.30 ± 27.51	4.00–98.00	24.00	[20.46–44.14]	b	A

Abbreviations: C.I., Confidence Interval; SD, standard deviation; T0, baseline; T1, 2 weeks.

**Table 10:** Descriptive statistics and intragroup/intergroup comparisons of questionnaire outcomes (Q1–Q10) across all follow-up time points (T1–T4) in the Trial and Control groups. * Means presenting at least one identical letter are not significantly different (*p* > 0.05). Intragroup analysis considered all the time frames of the study for the comparisons (lowercase letters), while intergroup analysis considered only Control vs. Trial comparison at each time frame (uppercase letters).

Question	Group	Time	Mean ± SD	Range	Median	C.I. (95%)	Intragroup (T1–T4) *	Intergroup (Control-Trial) *
Q1	Control (*n* = 20)	T1	1.65 ± 0.75	1.00–3.00	1.50	[1.32–1.98]	a	A
		T2	1.85 ± 0.75	1.00–3.00	2.00	[1.52–2.18]	a	A
		T3	1.90 ± 0.72	1.00–3.00	2.00	[1.58–2.22]	a	A
		T4	1.90 ± 0.72	1.00–3.00	2.00	[1.58–2.22]	a	A
	Trial (*n* = 20)	T1	2.55 ± 0.60	1.00–3.00	3.00	[2.28–2.82]	a	A
		T2	2.65 ± 0.49	2.00–3.00	3.00	[2.43–2.87]	a	A
		T3	2.65 ± 0.49	2.00–3.00	3.00	[2.43–2.87]	a	A
		T4	2.65 ± 0.49	2.00–3.00	3.00	[2.43–2.87]	a	A
Q2	Control (*n* = 20)	T1	1.45 ± 0.69	1.00–3.00	1.00	[1.15–1.75]	a	A
		T2	1.45 ± 0.69	1.00–3.00	1.00	[1.15–1.75]	a	A
		T3	1.45 ± 0.69	1.00–3.00	1.00	[1.15–1.75]	a	A
		T4	1.45 ± 0.69	1.00–3.00	1.00	[1.15–1.75]	a	A
	Trial (*n* = 20)	T1	0.45 ± 0.69	0.00–2.00	0.00	[0.15–0.75]	a	B
		T2	0.45 ± 0.69	0.00–2.00	0.00	[0.15–0.75]	a	B
		T3	0.45 ± 0.69	0.00–2.00	0.00	[0.15–0.75]	a	B
		T4	0.45 ± 0.69	0.00–2.00	0.00	[0.15–0.75]	a	B
Q3	Control (*n* = 20)	T1	0.60 ± 0.75	0.00–2.00	0.00	[0.27–0.93]	a	A
		T2	0.60 ± 0.75	0.00–2.00	0.00	[0.27–0.93]	a	A
		T3	0.60 ± 0.75	0.00–2.00	0.00	[0.27–0.93]	a	A
		T4	0.60 ± 0.75	0.00–2.00	0.00	[0.27–0.93]	a	A
	Trial (*n* = 20)	T1	1.70 ± 0.73	0.00–3.00	2.00	[1.38–2.02]	a	B
		T2	1.75 ± 0.79	0.00–3.00	2.00	[1.40–2.10]	a	B
		T3	1.75 ± 0.79	0.00–3.00	2.00	[1.40–2.10]	a	B
		T4	1.75 ± 0.79	0.00–3.00	2.00	[1.40–2.10]	a	B
Q4	Control (*n* = 20)	T1	4.00 ± 0.00	4.00–4.00	4.00	[4.00–4.00]	a	A
		T2	4.00 ± 0.00	4.00–4.00	4.00	[4.00–4.00]	a	A
		T3	4.00 ± 0.00	4.00–4.00	4.00	[4.00–4.00]	a	A
		T4	4.00 ± 0.00	4.00–4.00	4.00	[4.00–4.00]	a	A
	Trial (*n* = 20)	T1	4.00 ± 0.00	4.00–4.00	4.00	[4.00–4.00]	a	A
		T2	4.00 ± 0.00	4.00–4.00	4.00	[4.00–4.00]	a	A
		T3	4.00 ± 0.00	4.00–4.00	4.00	[4.00–4.00]	a	A
		T4	4.00 ± 0.00	4.00–4.00	4.00	[4.00–4.00]	a	A
Q5	Control (*n* = 20)	T1	1.70 ± 0.92	0.00–3.00	2.00	[1.30–2.10]	a	A
		T2	1.70 ± 0.92	0.00–3.00	2.00	[1.30–2.10]	a	A
		T3	1.70 ± 0.92	0.00–3.00	2.00	[1.30–2.10]	a	A
		T4	1.70 ± 0.92	0.00–3.00	2.00	[1.30–2.10]	a	A
	Trial (*n* = 20)	T1	2.70 ± 0.57	1.00–3.00	3.00	[2.45–2.95]	a	A
		T2	2.75 ± 0.55	1.00–3.00	3.00	[2.51–2.99]	a	A
		T3	2.75 ± 0.55	1.00–3.00	3.00	[2.51–2.99]	a	A
		T4	2.75 ± 0.55	1.00–3.00	3.00	[2.51–2.99]	a	A
Q6	Control (*n* = 20)	T1	0.45 ± 0.60	0.00–2.00	0.00	[0.19–0.71]	a	A
		T2	0.45 ± 0.60	0.00–2.00	0.00	[0.19–0.71]	a	A
		T3	0.45 ± 0.60	0.00–2.00	0.00	[0.19–0.71]	a	A
		T4	0.45 ± 0.60	0.00–2.00	0.00	[0.19–0.71]	a	A
	Trial (*n* = 20)	T1	1.10 ± 0.45	0.00–2.00	1.00	[0.90–1.30]	a	A
		T2	1.10 ± 0.45	0.00–2.00	1.00	[0.90–1.30]	a	A
		T3	1.10 ± 0.45	0.00–2.00	1.00	[0.90–1.30]	a	A
		T4	1.10 ± 0.45	0.00–2.00	1.00	[0.90–1.30]	a	A
Q7	Control (*n* = 20)	T1	2.25 ± 0.91	0.00–3.00	2.50	[1.85–2.65]	a	A
		T2	2.30 ± 0.86	0.00–3.00	2.50	[1.92–2.68]	a	A
		T3	2.30 ± 0.86	0.00–3.00	2.50	[1.92–2.68]	a	A
		T4	2.30 ± 0.86	0.00–3.00	2.50	[1.92–2.68]	a	A
	Trial (*n* = 20)	T1	2.80 ± 0.41	2.00–3.00	3.00	[2.62–2.98]	a	A
		T2	2.80 ± 0.41	2.00–3.00	3.00	[2.62–2.98]	a	A
		T3	2.80 ± 0.41	2.00–3.00	3.00	[2.62–2.98]	a	A
		T4	2.80 ± 0.41	2.00–3.00	3.00	[2.62–2.98]	a	A
Q8	Control (*n* = 20)	T1	1.70 ± 0.66	1.00–3.00	2.00	[1.41–1.99]	a	A
		T2	1.70 ± 0.66	1.00–3.00	2.00	[1.41–1.99]	a	A
		T3	1.70 ± 0.66	1.00–3.00	2.00	[1.41–1.99]	a	A
		T4	1.70 ± 0.66	1.00–3.00	2.00	[1.41–1.99]	a	A
	Trial (*n* = 20)	T1	1.85 ± 0.37	1.00–2.00	2.00	[1.69–2.01]	a	A
		T2	1.85 ± 0.37	1.00–2.00	2.00	[1.69–2.01]	a	A
		T3	1.85 ± 0.37	1.00–2.00	2.00	[1.69–2.01]	a	A
		T4	1.85 ± 0.37	1.00–2.00	2.00	[1.69–2.01]	a	A
Q9	Control (*n* = 20)	T1	0.00 ± 0.00	0.00–0.00	0.00	[0.00–0.00]	a	A
		T2	0.00 ± 0.00	0.00–0.00	0.00	[0.00–0.00]	a	A
		T3	0.00 ± 0.00	0.00–0.00	0.00	[0.00–0.00]	a	A
		T4	0.00 ± 0.00	0.00–0.00	0.00	[0.00–0.00]	a	A
	Trial (*n* = 20)	T1	0.00 ± 0.00	0.00–0.00	0.00	[0.00–0.00]	a	A
		T2	0.00 ± 0.00	0.00–0.00	0.00	[0.00–0.00]	a	A
		T3	0.00 ± 0.00	0.00–0.00	0.00	[0.00–0.00]	a	A
		T4	0.00 ± 0.00	0.00–0.00	0.00	[0.00–0.00]	a	A
Q10	Control (*n* = 20)	T1	0.10 ± 0.31	0.00–1.00	0.00	[0.00–0.24]	a	A
		T2	0.10 ± 0.31	0.00–1.00	0.00	[0.00–0.24]	a	A
		T3	0.10 ± 0.31	0.00–1.00	0.00	[0.00–0.24]	a	A
		T4	0.10 ± 0.31	0.00–1.00	0.00	[0.00–0.24]	a	A
	Trial (*n* = 20)	T1	0.00 ± 0.00	0.00–0.00	0.00	[0.00–0.00]	a	A
		T2	0.00 ± 0.00	0.00–0.00	0.00	[0.00–0.00]	a	A
		T3	0.00 ± 0.00	0.00–0.00	0.00	[0.00–0.00]	a	A
		T4	0.00 ± 0.00	0.00–0.00	0.00	[0.00–0.00]	a	A

Abbreviations: C.I., Confidence Interval; SD, standard deviation; T1, 2 weeks; T2, 1 month; T3, 3 months; T4, 6 months.

## Data Availability

Data produced and examined within this investigation can be obtained by contacting the corresponding author, provided the request is justified and appropriate.

## Supplementary Materials

The following supporting information can be downloaded at: https://www.mdpi.com/article/10.3390/dj14060383/s1, Table S1: CONSORT 2025 Checklist; Table S2: Patient-Reported Outcome Questionnaire Adapted from Basso et al. for the Comparative Clinical Evaluation of Essential Oil–Based Mouthwashes With and Without Alcohol; Table S3: Regression-based assessment of smoking status as a potential covariate.

## Abbreviations

The following abbreviations are used in this manuscript:

ABX	Antibacterial complex
aMMP-8	Activated matrix metalloproteinase-8
ASA	American Society of Anesthesiologists
BoP	Bleeding on Probing
CAL	Clinical Attachment Level
CHX	Chlorhexidine digluconate
CI	Confidence Interval
CONSORT	Consolidated Standards of Reporting Trials
CPC	Cetylpyridinium Chloride
FMBS	Full-Mouth Bleeding Score
FMPS	Full-Mouth Plaque Score
GBT	Guided Biofilm Therapy
MLSI	Modified Lobene Stain Index
PEG	Polyethylene Glycol
PPD	Probing Pocket Depth
Q1–Q10	Questionnaire items 1–10
RCT	Randomized Controlled Trial
REC	Gingival Recession
SD	Standard Deviation
T0	Baseline
T1	2-week follow-up
T2	1-month follow-up
T3	3-month follow-up
T4	6-month follow-up
