# Supplementary material for: Clinical Status and Salivary aMMP-8 Evaluation of 0.12% Chlorhexidine Versus MicroRepair® ABX Mouthwash in the Non-Surgical Management of Plaque-Induced Gingivitis: A Randomized Controlled Trial
Source: Dent J (Basel). 2026 Jun 20;14(6):383. doi: 10.3390/dj14060383 (PMC13297765; doi:10.3390/dj14060383)
Supplement: Supplementary file 1 [file dentistry-14-00383-s001.zip › dentistry-4323866-supplementary.pdf]

Table S1. CONSORT 2025 Checklist [1].

Section/topic	No	CONSORT 2025 checklist item description	Reported on page no.
**Title and abstract**			
Title and structured abstract	1a	Identification as a randomised trial	1
	1b	Structured summary of the trial design, methods, results, and conclusions	1
**Open science**			
Trial registration	2	Name of trial registry, identifying number (with URL) and date of registration	3,28
Protocol and statistical analysis plan	3	Where the trial protocol and statistical analysis plan can be accessed	3,6,7
Data sharing	4	Where and how the individual de-identified participant data (including data dictionary), statistical code and any other materials can be accessed	28
Funding and conflicts of interest	5a	Sources of funding and other support (eg, supply of drugs), and role of funders in the design, conduct, analysis and reporting of the trial	28
	5b	Financial and other conflicts of interest of the manuscript authors	28
**Introduction**			2,3
Background and rationale	6	Scientific background and rationale	3
Objectives	7	Specific objectives related to benefits and harms	
**Methods**			
Patient and public involvement	8	Details of patient or public involvement in the design, conduct and reporting of the trial	NR
Trial design	9	Description of trial design including type of trial (eg, parallel group, crossover), allocation ratio, and framework (eg, superiority, equivalence, non-inferiority, exploratory)	3,6
Changes to trial protocol	10	Important changes to the trial after it commenced including any outcomes or analyses that were not prespecified, with reason	NR
Trial setting	11	Settings (eg, community, hospital) and locations (eg, countries, sites) where the trial was conducted	3,4,7
Eligibility criteria	12a	Eligibility criteria for participants	4
	12b	If applicable, eligibility criteria for sites and for individuals delivering the interventions (eg, surgeons, physiotherapists)	5,6
Intervention and comparator	13	Intervention and comparator with sufficient details to allow replication. If relevant, where additional materials describing the intervention and comparator (eg, intervention manual) can be accessed	5,6, Table1
Outcomes	14	Prespecified primary and secondary outcomes, including the specific measurement variable (eg, systolic blood pressure), analysis metric (eg, change from baseline, final value, time to event), method of aggregation (eg, median, proportion), and time point for each outcome	4,5
Harms	15	How harms were defined and assessed (eg, systematically, non-systematically)	4,5,7,11,12
Sample size	16a	How sample size was determined, including all assumptions supporting the sample size calculation	5
	16b	Explanation of any interim analyses and stopping guidelines	NA
Randomisation:			
Sequence generation	17a	Who generated the random allocation sequence and the method used	6
	17b	Type of randomisation and details of any restriction (eg, stratification, blocking and block size)	6

			Reported on page no.
Allocation concealment mechanism	18	Mechanism used to implement the random allocation sequence (eg, central computer/telephone; sequentially numbered, opaque, sealed containers), describing any steps to conceal the sequence until interventions were assigned	6
Implementation	19	Whether the personnel who enrolled and those who assigned participants to the interventions had access to the random allocation sequence	6
Blinding	20a	Who was blinded after assignment to interventions (eg, participants, care providers, outcome assessors, data analysts)	6
	20b	If blinded, how blinding was achieved and description of the similarity of interventions	6
Statistical methods	21a	Statistical methods used to compare groups for primary and secondary outcomes, including harms	6,7
	21b	Definition of who is included in each analysis (eg, all randomised participants), and in which group	7, Tables 3-11
	21c	How missing data were handled in the analysis	7
	21d	Methods for any additional analyses (eg, subgroup and sensitivity analyses), distinguishing prespecified from post hoc	NR
**Results**			
Participant flow, including flow diagram	22a	For each group, the numbers of participants who were randomly assigned, received intended intervention, and were analysed for the primary outcome	7, Figure 1
	22b	For each group, losses and exclusions after randomisation, together with reasons	7, Figure 1
Recruitment	23a	Dates defining the periods of recruitment and follow-up for outcomes of benefits and harms	3,7
	23b	If relevant, why the trial ended or was stopped	NA
Intervention and comparator delivery	24a	Intervention and comparator as they were actually administered (eg, where appropriate, who delivered the intervention/comparator, how participants adhered, whether they were delivered as intended (fidelity))	5-7
	24b	Concomitant care received during the trial for each group	6
Baseline data	25	A table showing baseline demographic and clinical characteristics for each group	8, Table 2
Numbers analysed, outcomes and estimation	26	For each primary and secondary outcome, by group:<br>● the number of participants included in the analysis<br>● the number of participants with available data at the outcome time point<br>● result for each group, and the estimated effect size and its precision (such as 95% confidence interval)<br>● for binary outcomes, presentation of both absolute and relative effect size	8-24, Tables 3-11
Harms	27	All harms or unintended events in each group	7,11,12,28
Ancillary analyses	28	Any other analyses performed, including subgroup and sensitivity analyses, distinguishing pre-specified from post hoc	NR
**Discussion**			
Interpretation	29	Interpretation consistent with results, balancing benefits and harms, and considering other relevant evidence	25-28
Limitations	30	Trial limitations, addressing sources of potential bias, imprecision, generalisability, and, if relevant, multiplicity of analyses	27,28

Table S2. Patient-Reported Outcome Questionnaire Adapted from Basso et al. [2] for the Comparative Clinical Evaluation of Essential Oil–Based Mouthwashes With and Without Alcohol.

#	Question	None (0)	Slight (1)	Moderate (2)	Severe (3)	N/A* (4)
1	After repeated use of the mouthwash, did you notice less gum bleeding while brushing your teeth?	☐	☐	☐	☐	☐
2	Did you notice that your gums were less sore when using the mouthwash?	☐	☐	☐	☐	☐
3	Did you notice an improvement in your breath when using the mouthwash?	☐	☐	☐	☐	☐
4	If your teeth are sensitive to hot and cold, did the mouthwash improve this condition?	☐	☐	☐	☐	☐
5	Do your gums appear less red?	☐	☐	☐	☐	☐
6	Did the use of the mouthwash provide a general feeling of freshness in your mouth?	☐	☐	☐	☐	☐
7	Did the mouthwash make your teeth feel smoother and cleaner?	☐	☐	☐	☐	☐
8	Did you find the taste of the mouthwash pleasant?	☐	☐	☐	☐	☐
9	Did you notice an increase in tooth staining after using the mouthwash?	☐	☐	☐	☐	☐
10	Did the mouthwash cause any particular problems or discomfort?	☐	☐	☐	☐	☐

***N/A (Not Applicable):** symptom not present at baseline.

Table S3. Regression-based assessment of smoking status as a potential covariate.

Dependent variable	Predictor	Estimate	Standard error	t value	P value	Interpretation
Treatment group	Smoking status	-0.101	0.113	-0.892	0.375	Smoking status was not significantly associated with treatment allocation, indicating balanced smoking distribution between groups.
Smoking status	FMBS	-0.236	0.251	-0.941	0.350	Smoking status was not significantly associated with full-mouth bleeding score.
Smoking status	FMPS	-0.039	0.122	-0.321	0.749	Smoking status was not significantly associated with full-mouth plaque score.
Smoking status	PPD	-0.291	0.188	-1.545	0.126	Smoking status was not significantly associated with probing pocket depth.
Smoking status	CAL	-0.271	0.319	-0.850	0.398	Smoking status was not significantly associated with clinical attachment level.
Smoking status	REC	-0.778	0.425	-1.829	0.071	Smoking status showed a non-significant trend in relation to gingival recession.
Smoking status	MLSI	0.424	0.433	0.979	0.331	Smoking status was not significantly associated with extrinsic staining.
Smoking status	Time	<0.001	0.028	0.000	1.000	Smoking status was stable across follow-up time points.

Abbreviations: FMBS, Full-Mouth Bleeding Score; FMPS, Full-Mouth Plaque Score; PPD, Probing Pocket Depth; CAL, Clinical Attachment Level; REC, Gingival Recession; MLSI, Modified Lobene Stain Index. Smoking status was coded as a dichotomous variable, distinguishing non-smokers from light-to-moderate smokers. Heavy smokers, defined as individuals smoking more than 10 cigarettes per day, were excluded according to the eligibility criteria.

References

1. Hopewell, S.; Chan, A.W.; Collins, G.S.; Hróbjartsson, A.; Moher, D.; Schulz, K.F.; et al. CONSORT 2025 statement: Updated guideline for reporting randomised trials. *BMJ* **2025**, *388*, e081123.
2. Basso, M.; Nowakowska, J.; Bordini, G.; Corbella, S. Comparative clinical evaluation of two essential oil-based mouthwashes with and without alcohol. *Int J Dent Hyg* **2023**, *21*, 183–190.
